# The Antiviral Activity of Varenicline against Dengue Virus Replication during the Post-Entry Stage

**DOI:** 10.3390/biomedicines11102754

**Published:** 2023-10-11

**Authors:** Ching-Lin Lin, Yan-Tung Kiu, Ju-Ying Kan, Yu-Jen Chang, Ping-Yi Hung, Chih-Hao Lu, Wen-Ling Lin, Yow-Wen Hsieh, Jung-Yie Kao, Nien-Jen Hu, Cheng-Wen Lin

**Affiliations:** 1 Institute of Biochemistry, College of Life Sciences, National Chung Hsing University, Taichung 40227, Taiwan; lin921lin@gmail.com (C.-L.L.); biosjyk@gmail.com (J.-Y.K.); 2 Department of Medical Laboratory Science and Biotechnology, China Medical University, Taichung 404328, Taiwan; kytsally@gmail.com (Y.-T.K.); s0975782938@gmail.com (J.-Y.K.); h840528hank@yahoo.com.tw (P.-Y.H.); 3 The Ph.D. Program of Biotechnology and Biomedical Industry, China Medical University, Taichung 404328, Taiwan; u107311201@cmu.edu.tw; 4 Institute of Bioinformatics and Systems Biology, National Yang Ming Chiao Tung University, Hsinchu City 30010, Taiwan; chlu@nycu.edu.tw; 5 Department of Pharmacy, China Medical University Hospital, Taichung 404328, Taiwan; u101055002@gmail.com (W.-L.L.); yowenhsieh@gmail.com (Y.-W.H.); 6 School of Pharmacy, China Medical University, Taichung 404328, Taiwan; 7 Department of Medical Laboratory Science and Biotechnology, Asia University, Taichung 41354, Taiwan

**Keywords:** dengue virus, varenicline, antiviral, post entry, NS2B-NS3 protease, cleavage assay

## Abstract

Dengue virus (DENV) poses a significant global health challenge, with millions of cases each year. Developing effective antiviral drugs against DENV remains a major hurdle. Varenicline is a medication used to aid smoking cessation, with anti-inflammatory and antioxidant effects. In this study, varenicline was investigated for its antiviral potential against DENV. This study provides evidence of the antiviral activity of varenicline against DENV, regardless of the virus serotype or cell type used. Varenicline demonstrated dose-dependent effects in reducing viral protein expression, infectivity, and virus yield in Vero and A549 cells infected with DENV-1 and DENV-2, with EC50 values ranging from 0.44 to 1.66 μM. Time-of-addition and removal experiments demonstrated that varenicline had a stronger inhibitory effect on the post-entry stage of DENV-2 replication than on the entry stage, as well as the preinfection and virus attachment stages. Furthermore, cell-based trans-cleavage assays indicated that varenicline dose-dependently inhibited the proteolytic activity of DENV-2 NS2B-NS3 protease. Docking models revealed the formation of hydrogen bonds and van der Waals forces between varenicline and specific residues in the DENV-1 and DENV-2 NS2B-NS3 proteases. These results highlight the antiviral activity and potential mechanism of varenicline against DENV, offering valuable insights for further research and development in the treatment of DENV infection.

## 1. Introduction

Dengue virus (DENV) is a flavivirus transmitted by mosquitoes, with a single-stranded positive-sense RNA genome and four serotypes (DENV-1, -2, -3, and -4), along with other viruses such as Zika virus (ZIKV) and Japanese encephalitis virus (JEV) [1,2]. The DENV genome encodes a polyprotein of 10 proteins, including 3 structural proteins (capsid, premembrane, and envelope) and 7 nonstructural proteins (NS1, NS2A, NS2B, NS3, NS4A, NS4B, and NS5) [3]. The E protein, consisting of three domains, is embedded in the lipid membrane surrounding the capsid [4]. The post-translational proteolytic processing of nonstructural proteins is carried out by the NS2B-NS3 protease. RNA helicase, methyltransferase, and RNA-dependent RNA polymerase are among the individual nonstructural proteins that are released [5]. Since there is no licensed drug for DENV, the development of anti-DENV inhibitors is crucial for the control and treatment of DENV infection [6,7], which affects 50–100 million people worldwide each year, including cases of dengue hemorrhagic fever and fatalities. Recent studies have focused on finding potential antivirals that target the NS2b-NS3 protease, an essential enzyme complex in the DENV life cycle responsible for proteolytic and cleavage activities of the polyprotein [8]. NS2b stabilizes NS3pro and permits protein folding, and the NS2b-NS3pro cleaves multiple proteins during the virus life cycle. Phytochemicals including fatty acids, glucosides, terpenes, flavonoids, phenolics, chalcones, acetamides, and peptides have shown potential as inhibitors of DENV, with curcumin, quercetin, and myricetin acting as noncompetitive inhibitors of the NS2b-NS3 protease enzyme [9]. In silico and in vitro studies have provided ample phytochemical hits for the experimental assessment of their use as DENV protease inhibitors [10], indicating that natural product derivatives can be designed and synthesized to enhance specificity and efficacy toward these proteases.

Varenicline is a medication used to aid smoking cessation by binding to nicotinic acetylcholine receptors (nAChRs) in the brain, activating them as a full agonist on the α7 subunit and a partial agonist on the other subunits [11]. Recent research suggests that varenicline may have potential as a treatment for SARS-CoV-2 infection due to its high affinity for binding to the virus’s spike protein, which could inhibit the virus from replicating and binding to ACE2 [12,13]. Additionally, varenicline has been found to have anti-inflammatory and antioxidant effects, reducing inflammation and injury in mice following testicular torsion and inhibiting macrophage activity [14]. Varenicline is a modified alkaloid with a molecular structure similar to cytisine, another molecule used for smoking cessation in some European and Asian countries [15]. Cytisine is a partial agonist at nAChRs and has been shown to have antiviral activity against a variety of viruses, including influenza A, hepatitis B and C, and human immunodeficiency virus type 1 [16,17,18,19]. Recent studies have investigated the derivatives of cytisine, which have shown promising antiviral activity against DENV-1 and DENV-2, with some inhibiting virus attachment and entry stages, while others inhibiting the post-entry stage [20]. Thus, varenicline may exhibit similar antiviral abilities to cytisine against DENV, making it a potential candidate for future research on treating DENV infection.

In this study, we examined the antiviral activity and mechanism of varenicline against DENV in vitro to determine the structure–antiviral activity of varenicline with possible viral proteins. The antiviral activity and selectivity index of varenicline were examined; the mechanism of action of the derivatives on attachment, entry, and post-entry stages was also evaluated. Meanwhile, the potential interaction of varenicline with viral proteins was analyzed through further experimentation using trans-cleavage assays of NS2B-NS3 protease with the coexpression of the substrate in the cell, revealing possible antiviral actions. The results reveal the potential antiviral actions of varenicline and provide insights into its interaction with viral proteins.

## 2. Materials and Methods

### 2.1. Cells and Viruses

A viral stock of DENV-1 CMUH 2018-4, DENV-2 6681, ZIKV PRVABC59, and JEV T1P1 strains was prepared by amplifying them in African green monkey kidney epithelial Vero cells [21,22,23]. The anti-DENV activities and mechanisms of varenicline were tested on Vero cells and human lung epithelial A549 cells. Both cell lines were cultured in DMEM supplemented with 10% fetal bovine serum (FBS) and penicillin–streptomycin.

### 2.2. MTT Assay

The MTT assay was employed to determine the cytotoxicity of varenicline on Vero and A549 cells [21,22]. Vero or A549 cells were seeded in 96-well plates at a density of 5 × 10^3^ cells/well; treated with varenicline at concentrations of 0, 0.1, 10, 20, and 50 μM; and incubated for 96 h. Afterward, the cells were treated with an MTT solution and incubated for an additional 4 h at 37 °C. Cell viability (%) was calculated based on the reduction ability of MTT, and the concentration of varenicline that reduced cell viability by 50% (CC50) was determined using regression analysis.

### 2.3. Antiviral Assay with DENV-1, DENV-2, ZIKV, and JEV

To evaluate the anti-flavivirus activity of varenicline, cytopathic effect (CPE) reduction assays and in vitro infectivity assays were conducted on Vero and A549 cells [21,22]. Serial dilutions of DENV-1, DENV-2, ZIKV, and JEV stocks were prepared at various multiplicity of infections (MOIs), namely 0.005, 0.05, 0.01, 0.1, and 10. These dilutions were added to Vero cells and A549 cells to determine the appropriate MOI for observing CPE in a CPE reduction assay. Following this, a fixed quantity of these viruses was added into each well of Vero cells (DENV-1 at MOI 0.1, DENV-2 at MOI 0.005, ZIKV at MOIs 0.005 and 0.05, and JEV at MOI 0.005) and A549 cells (DENV-2 at MOI 0.05). The cells were simultaneously treated with varenicline at concentrations of 0.1, 1, and 10 μM. After 96 h of incubation, the DENV-induced cytopathic effect in infected/treated cells was observed and photographed under a microscope. Subsequently, the residual DENV-2 infectivity was determined by calculating the ratio of NS4B-positive cells to the total number of cells stained with DAPI (4′,6-diamidino-2-phenylindole) after double immunofluorescent staining assays (IFA) with anti-DENV NS4B antibodies (GeneTex, Inc., Hsinchu, Taiwan) and anti-rabbit IgG antibodies conjugated with AF555 (ThermoFisher, Waltham, MA, USA), as described in previous reports. The relative DENV infectivity by 50% (half-maximal effective concentration, EC50) was calculated using a computer program with the fitted line, and the selectivity index (SI) was derived by calculating the ratio of CC50 to EC50.

### 2.4. DENV-2 Yield Reduction Assay

Ten-fold serial dilutions of the media were added to Vero cells in 96-well plates. After 96 h of incubation, the number of cytopathic effects (CPEs) in each well was calculated. The dilution that resulted in 50% of the wells exhibiting CPEs was defined as the mean tissue culture infectious dose (TCID50). The virus yield was quantified by performing an end-point dilution assay, and the results are expressed as TCID50 per milliliter. The virus yield in the cultured media of mock-treated/infected cells was used as a reference point (100%) to determine the relative residual virus yield in the treated/infected cells.

### 2.5. Time-of-Addition and Removal Assays

The study used time-of-addition and removal assays to evaluate the inhibitory effects of varenicline on DENV replication at various stages, namely the attachment, entry, and post-entry stages. The assays consisted of three modes: cotreatment/removal, post-infection treatment/removal, and attachment modes. In the cotreatment/removal mode, Vero cells in 6-well plates were incubated with DENV-2 and the active compound simultaneously for one hour at 37 °C and then washed with PBS. In the post-infection treatment/removal mode, the cells were exposed to DENV-2 for a duration of two hours at 37 °C and subsequently washed with PBS, followed by a two-hour treatment with varenicline at specified concentrations, also at 37 °C. After this treatment period, the cells were again washed with PBS. In the attachment mode, the Vero cell monolayer in a 6-well plate was placed at 4 °C for two hours and cotreated with DENV-2 and varenicline at indicated concentrations for two hours at 4 °C. The treated/infected cells were then analyzed using an immune fluorescent staining assay to determine the residual DENV-2 infectivity. The inhibitory activity of varenicline during the attachment, entry, and post-entry stages of DENV replication was determined based on the residual infectivity.

### 2.6. Construction of DENV-2 NS2B-NS3 Gene and the FRET Substrate into the Expression Vector pcDNA3.1-HisC

The DENV-2 NS2BNS3 gene was amplified using reverse transcription polymerase chain reaction (RT-PCR) with the RNA genomes from DENV-2 strain 16681 as the template. Amplification was performed using a forward primer containing the HindIII restriction site and AVI tag sequence, and a reverse primer containing the foot-and-mouth disease virus 2A (F2A) peptide sequence and BamHI restriction site (Supplementary Table S1). After the PCR product and pcDNA3.1-HisC plasmid were digested with HindIII and BamHI, respectively, the DENV-2 NS2BNS3 gene was cloned into pcDNA3.1-HisC, thus generating a plasmid named pcDNA-DEN2_NS2BNS3 (Supplementary Figure S1A). To generate a fluorescence resonance energy transfer (FRET) substrate, namely a cyan fluorescent protein (CFP)–cleavage site Thr-Arg-Arg-Gly (trrg)-yellow fluorescent protein (YFP) (CFP-trrg-YFP), cDNA fragments of CFP-trrg and trrg-YFP were separately amplified with PCR using specific primer pairs (Supplementary Table S1). The templates used for PCR were previously reported in our study [24]. The reverse primer for the CFP-trrg fusion gene included the Thr-Arg-Arg-Gly cleavage site sequence at the 3′ end, while the forward primer for the trrg-YFP fusion gene contained the Thr-Arg-Arg-Gly cleavage site sequence at the 5′ end (Supplementary Table S1). The PCR products were then ligated using a Gibson assembly kit, resulting in an in vitro ligated fragment of CFP-trrg and trrg-YFP. This ligated fragment was subsequently cloned into pcDNA3.1-HisC, yielding the plasmid pcDNA-CFP-trrg-YFP (Supplementary Figure S1B).

### 2.7. Trans-Cleavage Assays with a CFP-YFP FRET Substrate Using DENV-2 NS2BNS3

To generate a CFP-trrg-YFP-expressing HEK293T cell line, the cells transfected with pcDNA-CFP-trrg-YFP underwent G418 selection. These cells were then seeded in a 24-well plate at a density of 2.5 × 10^4^ cells per well and allowed to adhere overnight. Transfection was performed using 50 ng of pcDNA-DEN2_NS2BNS3 plasmid and a jetPRIME transfection kit (Polyplus, Illkirch, France) according to the manufacturer’s instructions. After a 4 h incubation, the transfected cells were treated with specified concentrations of varenicline for 24 h. Following the treatment, the cells were rinsed with PBS and then exposed to a 1X passive lysis buffer, a specialized formulation designed to facilitate the rapid lysis of cultured adherent cells (Promega, Madison, WI, USA). The cells were then incubated in the dark at room temperature while shaking for 15 min. Subsequently, the cell lysates were transferred into a 96-well black plate. FRET signals were detected using a SpectraMax Multi-Mode Microplate Reader (Molecular Devices, San Jose, CA, USA) with emission–extension wavelengths set at 436–528n, 434–474nm, and 440–485nm. To obtain the FRET intensity in each well, the background signal from mock cells was subtracted. The activity of DENV-2 NS2BNS3 was normalized by subtracting the FRET signal in mock-transfected CFP-trrg-YFP-expressing cells and was presented as enzymatic activity, with 100% representing the baseline activity. Additionally, the inhibitory ability of varenicline was assessed by measuring the relative decrease in DENV-2 NS2BNS3 activity in cells treated with the indicated concentrations of varenicline.

### 2.8. Molecular Docking

The crystal structures of DENV-1, DENV-2, ZIKV, and JEV NS2B-NS3 proteases were chosen as the target proteins for virtual screening. The catalytically active form of these proteases (PDB ID: 3L6P, 2FOM, 5H4I, and 4R8T) was utilized. The binding site or pocket in the NS2B-NS3 protease was prepared by selecting residues within 8Å of His51, Asp75, and Ser135 as the receptor binding site. The corresponding residues in the other three flaviviruses were then obtained. To evaluate the prepared binding pocket, a multiple sequence alignment was performed using the Clustal W tool. The NS2B-NS3 protease sequences of the four flaviviruses were aligned, and the conserved region of the NS2B-NS3 protease structure was used for evaluation. To investigate the potential binding of varenicline to the active site of the NS2B-NS3 proteases, the iGEMDOCK software (version 2.1) (BioXGEM, Hsinchu, Taiwan), a widely used docking program known for accurate predictions, was employed. The docking process involved three types of interactions: electrostatic forces, hydrogen bonding, and van der Waals forces. The predicted conformers of varenicline were selected based on these interactions, which were evaluated using a scoring function that combined a simple empirical scoring approach and a pharmacophore-based scoring function.

### 2.9. Statistical Analysis

The data obtained from the experiments were analyzed using a one-way ANOVA, followed by Scheffe’s post hoc test, using SPSS 12.0 software (SPSS, Inc. Chicago, IL, USA). A *p*-value less than 0.05 was considered statistically significant.

## 3. Results

### 3.1. Identification of Varenicline as the Inhibitor against DENV, Not JEV or ZIKV

**Figure 1:**
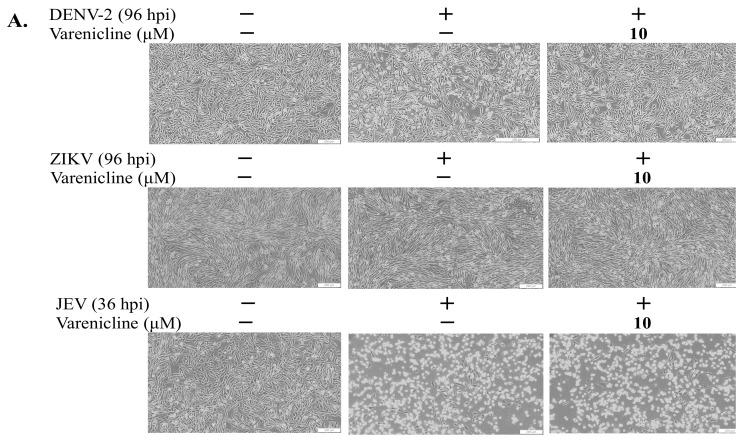

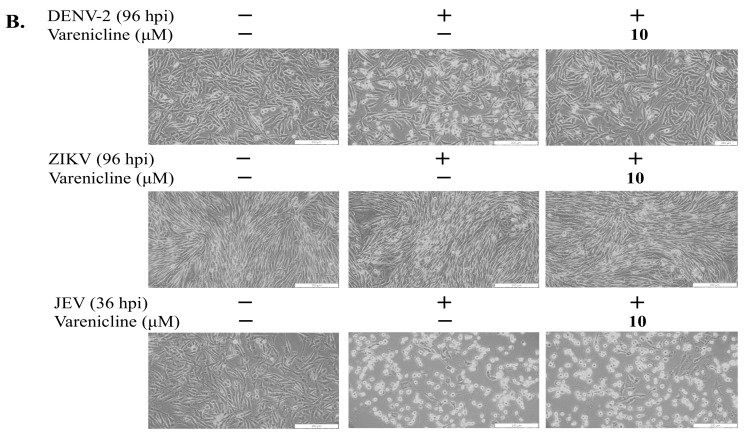
Inhibitory effect of varenicline on cytopathic effect in the infected cells with DENV-2, ZIKV, and JEV. Vero cells were infected with DENV-2, ZIKV, and JEV at a MOI of 0.005, respectively. The cells were treated with varenicline at a concentration of 10 μM immediately after infection. The inhibitory effect of varenicline on virus-induced cytopathic effects was observed and recorded using an inverted microscope at low (**A**) and high (**B**) magnifications after infection; scale bar: 200 μm.

**Figure 2:**
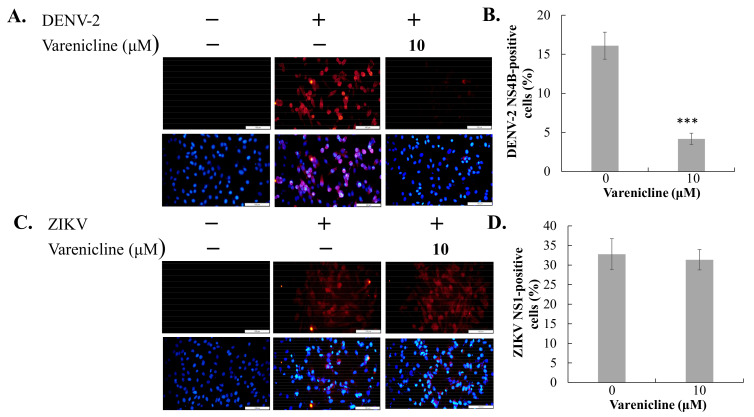
Inhibitory effect of varenicline on viral protein expression in infected cells with DENV-2 and ZIKV. Vero cells were infected with DENV-2 (**A**,**B**) and ZIKV (**C**,**D**) at an MOI of 0.005, respectively. The cells were treated with varenicline at a concentration of 10 μM immediately after infection. DENV-2 NS4B or ZIKV NS1 expression was examined 96 h after infection using IFA with anti-DENV-2 NS4B or anti-ZIKV NS1 antibodies, along with secondary antibodies conjugated with AF555. Cell nuclei were subsequently stained with DAPI. Merged images from IFA and DAPI staining were generated to determine the percentage of DENV-2 or ZIKV positivity normalized by total nuclei. Data were collected from three independent experiments; ***, *p*-value < 0.001 compared with mock-treated/infected cells; scale bar: 100 μm.

To determine the most effective concentrations of varenicline that have minimal toxicity, the researchers conducted MTT assays to evaluate its cytotoxicity on Vero and A549 cells. The CC50 value of varenicline for both cell types was found to be greater than 50 μM (Supplementary Figure S2). Based on this information, a maximum concentration of 20 μM varenicline was selected for subsequent antiviral assays. For screening the antiviral activity against flaviviruses (DENV-2, ZIKV, and JEV), a concentration of 10 μM of varenicline was utilized (Figure 1 and Figure 2). In the cytopathic effect reduction assay, the 10 μM concentration of varenicline significantly inhibited cytopathic changes in cells infected with DENV-2 but did not show the same effect against ZIKV or JEV (Figure 1 and Figure S3). In the immunofluorescent staining assay, the 10 μM varenicline concentration notably reduced the expression of viral proteins in DENV-2-infected cells but not in ZIKV-infected cells (Figure 2). These results demonstrate the antiviral activity of varenicline against DENV-2, as evidenced by the significant reduction in cytopathic effects and infectivity in Vero cells. Therefore, further antiviral activity analysis of varenicline against DENV-2 was conducted using two different cell lines.

### 3.2. Antiviral Activity of Varenicline against DENV-1 and DENV-2

**Figure 3:**
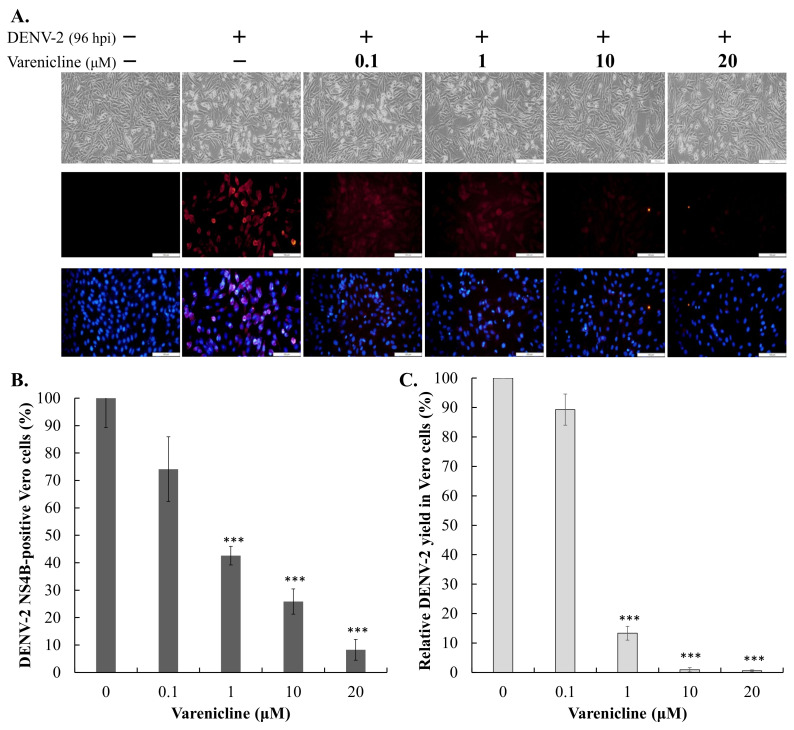
Concentration-dependent inhibition of varenicline on DENV-2 infectivity in Vero cells. The cells were infected with DENV-2 at an MOI of 0.005 and immediately treated with varenicline at the indicated concentrations. The inhibitory effect of varenicline on virus-induced cytopathic effects was observed and recorded using an inverted microscope 96 h after infection ((**A**), top). The treated/infected cells were then subjected to immunofluorescence assay (IFA) using anti-DENV-2 NS4B antibodies and secondary antibodies conjugated with AF555 ((**A**), middle and bottom). DENV-2 infectivity was determined by calculating the ratio of NS4B-positive cells to the total number of nuclei stained with DAPI (**B**). The virus yield was quantified using the TCID50 assay, and the relative residual virus yield in the treated/infected cells was calculated based on the virus yield in a cultured medium of mock-treated/infected cells, which was used as a reference (100%) (**C**). Data were collected from three independent experiments; ***, *p*-value < 0.001 compared with mock-treated/infected cells; scale bar: 200 μm ((**A**), top) and 100 μm ((**A**), middle and bottom).

**Figure 4:**
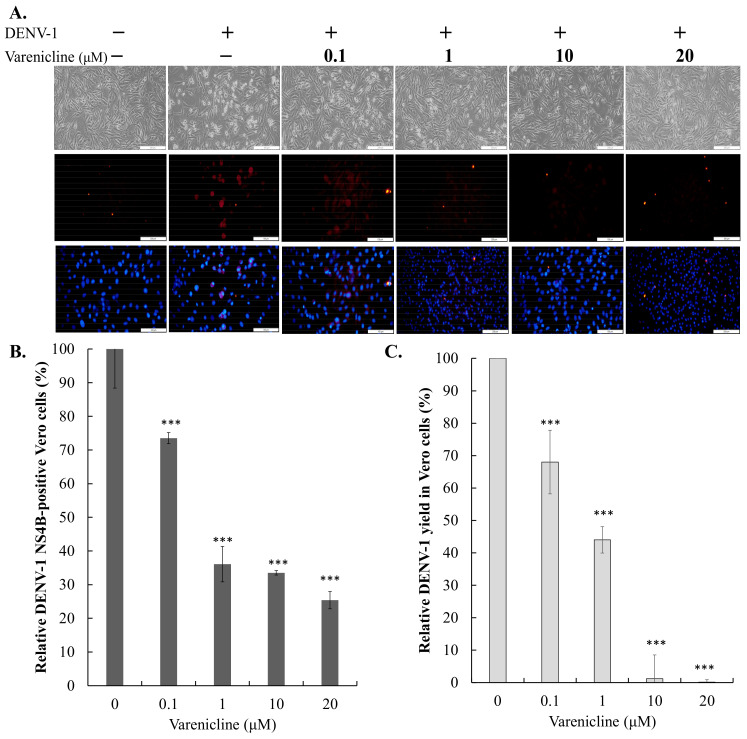
Concentration-dependent inhibition of varenicline on DENV-1 infectivity in Vero cells. Cells were infected with DENV-1 and treated with varying concentrations of varenicline. The inhibitory effect on virus-induced cytopathic effects was observed using an inverted microscope ((**A**), top). Immunofluorescence assay (IFA) was conducted to assess DENV-1 infectivity by measuring the ratio of NS4B-positive cells to the total number of nuclei stained with DAPI ((**A**) middle and bottom, (**B**)). Virus yield was determined using the TCID50 assay, and the relative residual virus yield in treated/infected cells was calculated relative to mock-treated/infected cells (**C**). Data were collected from three independent experiments; ***, *p*-value < 0.001 compared with mock-treated/infected cells; scale bar: 200 μm ((**A**) top) and 100 μm ((**A**) middle and bottom).

To determine the half-maximal effective concentration (EC50) of varenicline against DENV-2, serial dilutions of varenicline were used to treat Vero and A549 cells infected with DENV-2 (Figure 3 and Figure 4). Varenicline exhibited dose-dependent effects by reducing cytopathic effects and DENV-2 protein expression in the infected cells. Furthermore, varenicline demonstrated a meaningful inhibition of infectivity and virus yield, with EC50 values of 0.73 μM for inhibiting infectivity in Vero cells and 1.66 μM for inhibiting infectivity in A549 cells. Additionally, the EC50 values for reducing virus yield were 0.47 μM in Vero cells and 1.66 μM in A549 cells. On the other hand, the antiviral activity of varenicline against DENV-1 was also evaluated in Vero cells using the aforementioned assays (Figure 4). Varenicline displayed dose-dependent effects by reducing cytopathic effects and DENV-1 protein expression in the infected cells, as well as by inhibiting infectivity and reducing virus yield. The EC50 values for inhibiting infectivity and reducing virus yield in Vero cells were determined to be 0.81 μM and 0.44 μM, respectively. These findings indicate that varenicline has significant antiviral activity against DENV, regardless of the virus serotype or cell type used, highlighting its broad-spectrum effectiveness.

### 3.3. Antiviral Actions of Varenicline during the Replication Cycle of DENV

**Figure 5:**
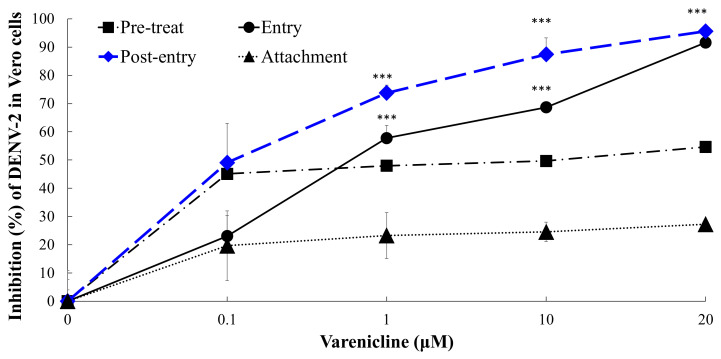
The antiviral actions of varenicline against DENV were analyzed using time-of-addition and removal assays in vitro. Vero cells were infected with DENV-2, and varenicline treatment was applied at various stages: pretreatment, attachment, entry, and post-entry. After washing, the cells were incubated for 4 days at 37 °C in a CO_2_ incubator. At 96 h after infection, the cells were assayed using immunofluorescence staining with anti-DENV NS4B antibodies and secondary antibodies conjugated with AF555. DENV infectivity was determined by calculating the ratio of NS4B-positive cells to the total number of nuclei stained with DAPI. The degree of infectivity inhibition was calculated based on the decrease in the percentage of NS4B-positive cells after treatment. Data were collected from three independent experiments; ***, *p*-value < 0.001 compared with the attachment mode.

To gain insight into the antiviral action of varenicline on different stages of DENV replication, a time-of-addition and removal experiment was conducted. This experiment involved treating cells with varenicline at specific time points during the DENV replication cycle: pretreatment of cells (2 h before infection, followed by washing), entry stage (during the second hour of infection, followed by washing), post-entry stage (2 h after infection, followed by washing), and receptor binding stage (during virus attachment at 4 °C, followed by washing) (Figure 5). In both the entry and post-entry modes, varenicline exhibited dose-dependent effects on virus infectivity, as determined by the reduction in the percentage of DENV-2 NS4B-positive cells compared to DAPI-positive nuclei. The results indicated that varenicline had a stronger inhibitory effect on the post-entry stage (EC50 < 0.1 μM) compared to the entry stage (EC50 = 0.86 μM), preinfection (pretreatment mode) (EC50 = 3.24 μM), and virus attachment (EC50 > 20 μM). These findings suggest that treatment with varenicline resulted in a notable reduction in DENV RNA translation, replication, and polyprotein processing steps during DENV-2 replication. The results highlight the ability of varenicline to interfere with crucial stages of the viral replication cycle, particularly the post-entry stage, contributing to its antiviral activity against DENV-2.

### 3.4. Inhibitory Activity of Varenicline on DENV NS2B-NS3 Protease Mediated the Proteolysis of FRET Substrates

**Figure 6:**
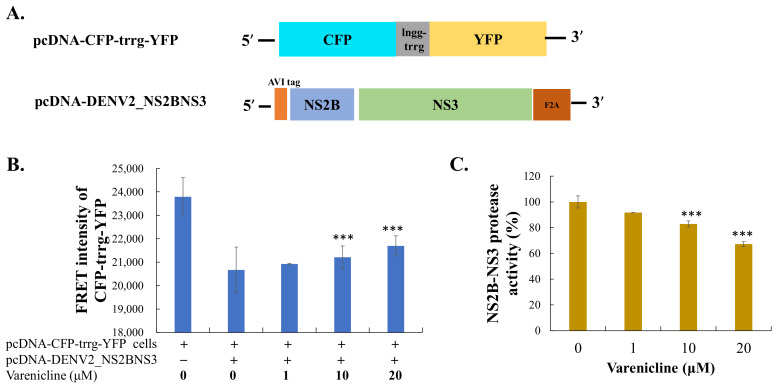
A trans-cleavage FRET assay was used to quantify the inhibitory activity of varenicline on DENV-2 NS2B-NS3 protease. The cartoon figure (**A**) represents the trans-cleavage FRET substrate employed for this assay, which is acted upon by DENV-2 NS2B-NS3 protease. The cells were transiently coexpressing CFP-lngg-trrg-YFP and DENV-2 NS2B-NS3 protease. These cells were treated with varenicline for a duration of 24 h. The FRET signal (426–528 nm) emitted by the cells was then measured (**B**). The FRET signal obtained from mock-treated cells was considered to represent 100% of the DENV-2 NS2B-NS3 protease activity. By comparing the FRET signal of the treated cells with that of the mock-treated cells, the relative residual enzymatic activity in the treated cells was calculated (**C**). Data were collected from three independent experiments. ***, *p*-value < 0.001 compared with mock-treated/infected cells.

One crucial step in the early post-entry stage of DENV replication is the proteolytic processing of the viral polyprotein by the DENV NS2B-NS3 protease, which was recognized as the potential target for varenicline. To validate the prediction, the inhibitory effect of varenicline on the proteolysis mediated by the NS2B-NS3 protease was evaluated (Figure 6). The FRET substrate CFP-trrg-YFP was developed and used in a trans-cleavage assay with the DENV-2 NS2B-NS3 protease. Treatment with varenicline resulted in a dose-dependent increase in FRET signals from CFP-trrg-YFP, indicating the inhibition of the enzymatic activity of the DENV-2 NS2B-NS3 protease, with an IC50 value greater than 20 μM (Figure 6). The findings revealed that varenicline significantly inhibited the enzymatic activity of the DENV NS2B-NS3 protease.

### 3.5. In Silico Modeling of Varenicline Interaction with DENV Proteins

**Figure 7:**
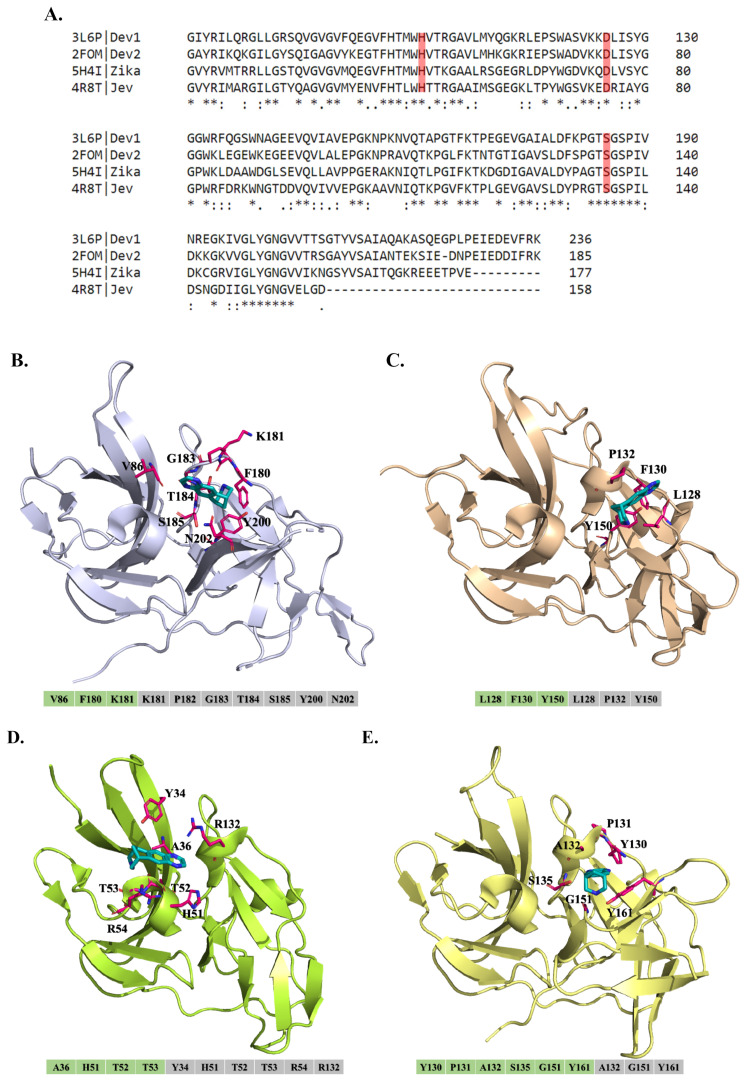
Sequence alignment and 3-D models of flaviviral NS2B-NS3 proteases for molecular docking with varenicline. The alignment displays the binding sites in NS2B-NS3 protease from DENV-1, DENV-2, JEV, and ZIKV, with the PDB codes of each virus on the left and the corresponding residue identifiers in NS2B-NS3 protease on the right. The catalytic triad within the active site of the NS3 serine protease consists of three specific amino acid residues H51, D75, and S135 that are highlighted in red (**A**). The figure also presents the molecular docking predictions of varenicline binding to the active sites within the proteases from DENV-1, DENV-2, JEV, and ZIKV (**B**–**E**). The cartoon figures illustrate the protease structures for each virus, namely DENV-1 (**B**), DENV-2 (**C**), JEV (**D**), and ZIKV (**E**). Varenicline is represented in a deeper cyan color, and the interactive residues are depicted as hot-pink sticks. Additionally, colored bars beneath each NS2B-NS3 protease indicate hydrogen bonding (light green) and van der Waals forces (grey).

To investigate the mechanism of varenicline against DENV, docking algorithms were employed to assess its potency against flavivirus proteases. Four protein structures of the NS2B-NS3 protease active form (PDB ID: 3L6P, 2FOM, 5H4I, and 4R8T) were selected as target proteins, generating three-dimensional (3-D) models to depict the interactive residues and binding forces between proteases and varenicline (Figure 7A). In the DENV-1 predicted model, varenicline was found to form hydrogen bonds with residues V86, F180, and K181. Additionally, residue K181 exhibited van der Waals forces, suggesting its potential importance in the interaction between varenicline and DENV-1 NS2B-NS3 protease (Figure 7B). In the DENV-2 model, varenicline formed hydrogen bonds with residues L128, F130, and Y150, while residues L128 and Y150 also displayed van der Waals forces (Figure 7C). On the other hand, the interactive residues between JEV or ZIKV and DENV showed different patterns. In the JEV binding model, residues A36, H51, T52, and T53 interacted through hydrogen bonds, while Y34, R54, and R132 only displayed van der Waals forces. Additionally, residues H51, T52, and T53 formed van der Waals forces with varenicline (Figure 7D). In the ZIKV prediction model, multiple hydrogen bond forces were observed involving residues Y130, P131, A132, S135, G151, and Y161, with the latter three residues also exhibiting van der Waals forces (Figure 7E). The different docking models revealed distinct binding modes, but they also identified common interactive residues. These shared interactions may account for the higher potency of varenicline against DENV-1 and DENV-2 than against JEV or ZIKV, as observed in the antiviral activity results against DENV (Figure 1 and Figure 2). The docking studies provide valuable insights into the potential binding interactions between varenicline and DENV proteases, which aligns with the findings that varenicline specifically inhibits the post-entry stage of DENV infection by involving the inhibitory effect on NS2B-NS3 protease activity.

## 4. Discussion

This study highlights varenicline, a medication commonly used for smoking cessation and treating dry eye disease, as a potential candidate for repurposing in the treatment of DENV infection. The study demonstrates that varenicline exhibits anti-DENV activity in a manner independent of the virus and cell type, with EC50 values below 2 μM and a selectivity index (SI) greater than 30 (Figure 1, Figure 2, Figure 3, Figure 4 and Figure S3). These findings suggest the potential of varenicline as a repurposed drug for combating DENV infection. The field of drug repurposing has garnered significant attention in the quest for new clinical applications of approved or failed drugs. In the case of DENV, various therapeutic molecules from different drug classes, such as antidiabetic drugs, anticholesteremic drugs, antihistamines, antipsychotic drugs, anticancer drugs, antibiotics, antiparasitic agents, and antimalarial drugs, have been repurposed [25,26,27]. N-acetyl cysteine (NAC), used for acetaminophen toxicity, shows promise in treating DENV-induced liver injury. NAC reduces viral particles, improves symptoms, and protects the liver by enhancing antiviral responses and maintaining antioxidant balance [28]. Considering the previous successes in drug repurposing for DENV, varenicline emerges as a potential candidate for further investigation in the treatment of DENV infection. Its demonstrated anti-DENV activity, and the well-established safety profile of varenicline warrant further evaluation of its efficacy and safety in the context of DENV infection.

Varenicline effectively inhibits DENV replication in the post-entry stage (Figure 5). Cell-based trans-cleavage assays utilizing DENV NS2B-NS3 protease were conducted, and their results reveal that varenicline inhibits the enzymatic activity of the DENV-2 NS2B-NS3 protease in a dose-dependent manner (Figure 6). Through the use of docking algorithms, it was found that varenicline specifically interacts with key residues in the DENV-1 and DENV-2 protease models, explaining its increased potency against DENV compared with other flaviviruses such as JEV and ZIKV (Figure 7). Four protein structures of the NS2B-NS3 protease active form (PDB ID: 3L6P, 2FOM, 5H4I, and 4R8T) were selected as target proteins, generating three-dimensional (3-D) models to depict the interactive residues and binding forces between proteases and varenicline (Figure 7). The shared interactions may account for the higher potency of varenicline against DENV-1 and DENV-2 than against JEV or ZIKV, as observed in the antiviral activity results against DENV (Figure 1 and Figure 2). The docking studies provide valuable insights into the potential binding interactions between varenicline and DENV proteases, which aligns with the findings that varenicline inhibits DENV NS2B-NS3 protease, associated with the involvement in the post-entry stage of DENV infection (Figure 5 and Figure 6). Previous research has also focused on the NS3 protease as a target for drug repurposing against DENV [29]. By merging the pharmacophore alignment model with known NS3 protease inhibitors, researchers have identified potential anti-DENV candidates such as boceprevir, telaprevir, and asunaprevir. Subsequent experiments confirmed the inhibitory effects of telaprevir and asunaprevir on DENV replication [29]. These findings emphasize the potential of repurposing existing drugs for the treatment of DENV infection.

Varenicline might not only inhibit the NS2B-NS3 protease activity of DENV but also exhibit inhibitory activity on other viral proteins or cellular factors related to the post-entry stage of DENV replication. This broader targeting of DENV proteins or host cellular factors may explain why the IC50 value for varenicline on NS2B-NS3 protease activity is higher than the EC50 value for DENV infectivity and virus yield (Figure 3, Figure 4 and Figure S2). Additionally, molecular simulations have revealed that a specific region of the SARS-CoV-2 spike protein can bind to nicotinic acetylcholine receptors (nAChRs) [30], and in vitro and in vivo studies have shown that varenicline tartrate can reduce SARS-CoV-2 infectivity and replication [31]. These findings indicate that varenicline may have the ability to interact with additional viral proteins and/or host cellular factors, potentially leading to the inhibition of DENV infection.

## 5. Conclusions

In conclusion, this study suggests that varenicline, a medication commonly used for smoking cessation, exhibits an effective anti-DENV activity via the potent inhibition of DENV replication during the post-entry stage. It reduces the NS2B-NS3 protease-mediated proteolysis process in viral replication. These findings highlight the potential of repurposing varenicline for the treatment of DENV infection.

## Data Availability

Not applicable.

## Supplementary Materials

The following supporting information can be downloaded at: https://www.mdpi.com/article/10.3390/biomedicines11102754/s1, Figure S1: The cytotoxicity of varenicline on Vero and A549 cells assessed using the MTT assay; Figure S2: Inhibitory effect of varenicline on cytopathic effect in the infected cells with ZIKV; Figure S3: The concentration-dependent inhibition of varenicline on DENV-2 infectivity in A549 cells; Figure S4: The concentration-dependent inhibition of varenicline on DENV-2 infectivity in A549 cells. Table S1: Primers used for cloning DENV-2 NS2B-NS3 gene and FRET substrates into the expression vector pcDNA3.1-HisC.
