# Supplementary material for: The Antiviral Activity of Varenicline against Dengue Virus Replication during the Post-Entry Stage
Source: Biomedicines. 2023 Oct 11;11(10):2754. doi: 10.3390/biomedicines11102754 (PMC10604274; doi:10.3390/biomedicines11102754)
Supplement: Supplementary file 1 [file biomedicines-11-02754-s001.zip › biomedicines-2615733-supplementary.pdf]

## Supplementary materials and Figures

**Supplementary Table 1**. Primers used for cloning DENV-2 NS2B-NS3 gene and FRET substrates into the expression vector pcDNA3.1-HisC

| Primer names | Primer sequences |
|---|---|
| DEN2-NS2b-HindIII AVI tag For | 5’-ttaagcttaccatgggcctgaacgacatcttcgaggcgcagaagattgaatggcatgaaggtggcggtggcagcagctggccattaaatgaggcta-3’ |
| DEN2-protease-F2A-BamH1-Rev | 5’ttggatccgggcccggggttggactcaacgtctcctgccaacttgagcaaatcaaagttctttcttccggctgcaaattcctta-3 |
| CFP-BamHI-For | 5’-ccaggatccatggttagcaagggcgaggaac-3’ |
| CFP-lngg-trrg-YFP-Rev | 5’-catcccacgacgagtgtatgcccctccgttaaggctggttttgtacagctcgt-3' |
| CFP-lngg-trrg-YFP-For | 5’-agccttaacggaggggcatacactcgtcgtgggatggttagcaaaggcgaaga-3' |
| YFP-XhoI-Rev | 5’-agactcgagtttgtacagttcgtccataccca-3’ |

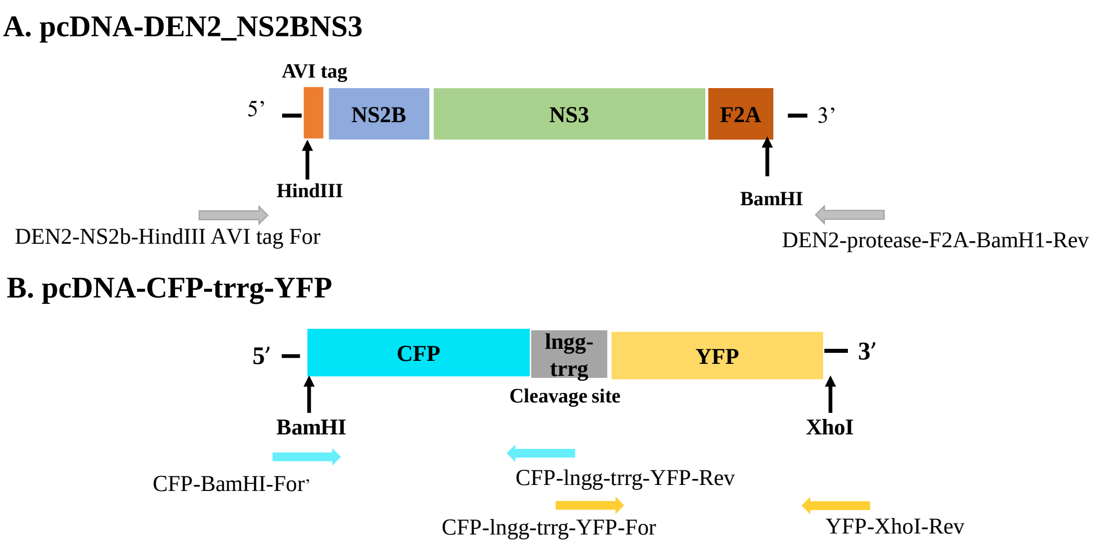


**Supplementary Figure 1. Construction and cloning DENV-2 NS2B/NS3, and the FRET substrate CFP-trrg-YFP into pcDNA3.1-HisC plasmid.** The DENV-2 NS2BNS3 gene was amplified using reverse transcription polymerase chain reaction (RT-PCR) with RNA genomes from DENV-2 strain 16681 as the template. The amplified product was then cloned into pcDNA3.1-HisC plasmid, resulting in the creation of a plasmid named pcDNA-DEN2_NS2BNS3 (A). To generate fluorescence resonance energy transfer (FRET) substrates, cDNA fragments of CFP-lngg-trrg and lngg-trrg-YFP were separately amplified by PCR and ligated together. The ligated fragment was cloned into pcDNA3.1-HisC, yielding the plasmid pcDNA-CFP-trrg-YFP (B).

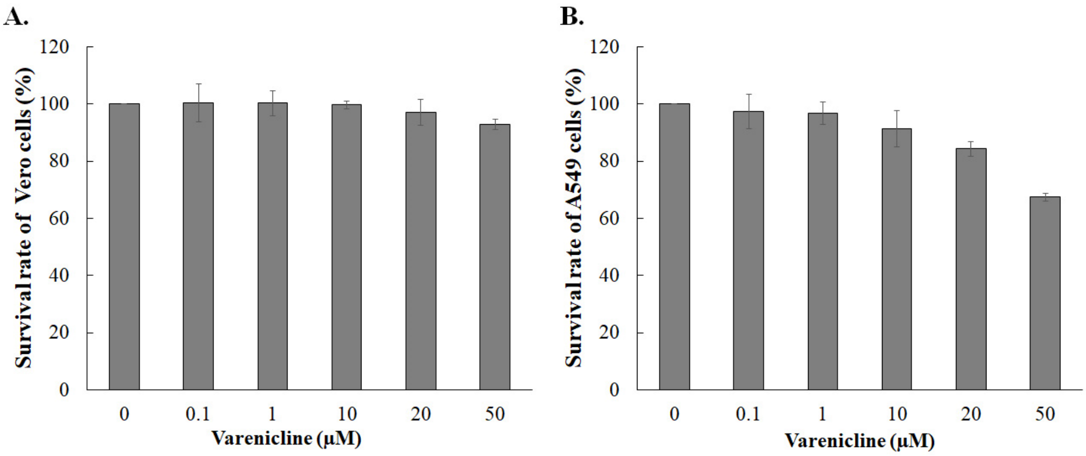


**Supplementary Figure 2. The cytotoxicity of varenicline on Vero and A549 cells assessed using the MTT assay.** Vero (A) and A549 (B) cells were treated with different concentrations of varenicline (0, 0.1, 10, 20, and 50 μM) and incubated for 96 hours. After the incubation period, the cells were exposed to MTT solution and incubated for an additional 4 hours at 37 °C. Cell viability (%) was determined by measuring the reduction of MTT.

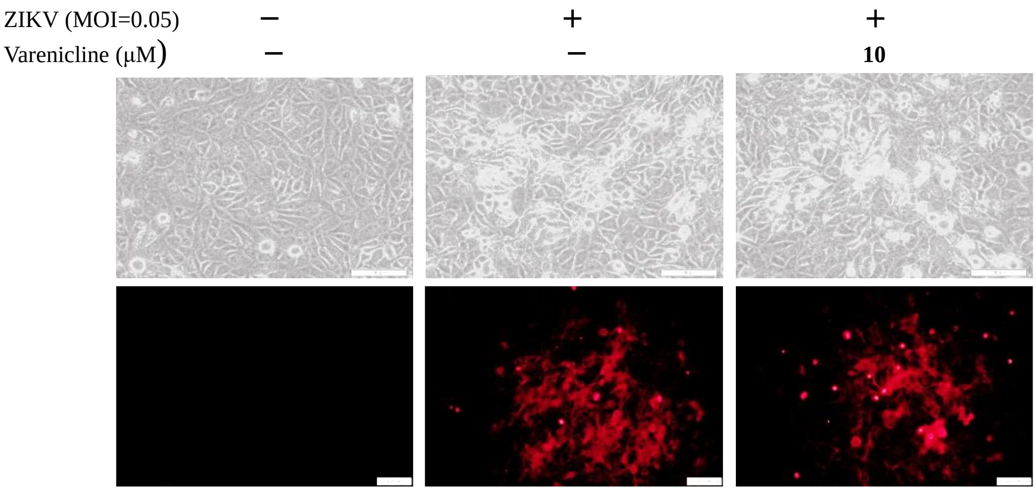


**Supplementary Figure 3**. Inhibitory effect of varenicline on cytopathic effect in the infected cells with ZIKV. Vero cells were infected with ZIKV at a MOI of 0.05, treated with varenicline at a concentration of 10 μM immediately after infection. The inhibitory effect of varenicline on virus-induced cytopathic effects was observed and recorded using an inverted microscope 96 hours post infection. ZIKV NS1 expression was examined using IFA with anti-ZIKV NS1 antibodies, along with secondary antibodies conjugated with AF555. Scale bar, 100 μm

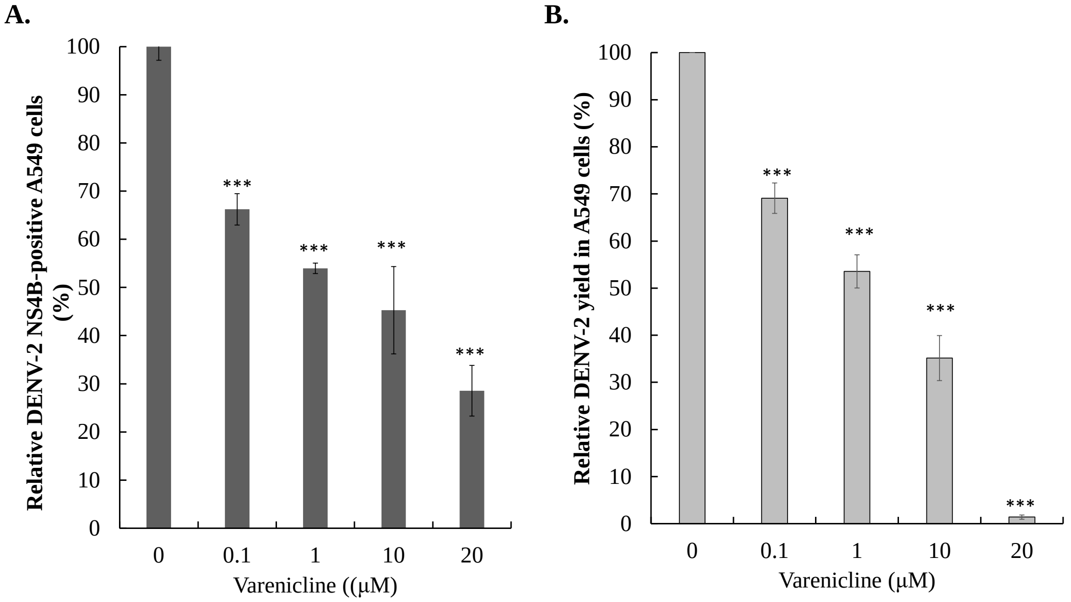


**Supplementary Figure 4. The concentration-dependent inhibition of varenicline on DENV-2 infectivity in A549 cells.** The cells were infected with DENV2 at a multiplicity of infection (MOI) of 0.05 and immediately treated with varying concentrations of varenicline. The inhibitory effect of varenicline on virus-induced cytopathic effects was observed and recorded using an inverted microscope 96 hours post-infection. Immunofluorescence assay (IFA) was performed on the treated/infected cells using anti-DENV-2 NS4B antibodies and secondary antibodies conjugated with AF555. The ratio of NS4B-positive cells to the total number of nuclei stained with DAPI was calculated to determine DENV-2 infectivity (A). Additionally, the virus yield was quantified using the TCID50 assay, and the relative residual virus yield in the treated/infected cells was calculated based on the virus yield in the cultured media of mock-treated/infected cells, which was used as a reference (100%) (B). Data were collected from three independent experiments. ***, p value<0.001 compared with mock-treated/infected cells.
